# pH-Responsive Resveratrol-Loaded Electrospun Membranes for the Prevention of Implant-Associated Infections

**DOI:** 10.3390/nano10061175

**Published:** 2020-06-16

**Authors:** Irene Bonadies, Francesca Di Cristo, Anna Valentino, Gianfranco Peluso, Anna Calarco, Anna Di Salle

**Affiliations:** 1 Institute for Polymers, Composites and Biomaterials (IPCB-CNR) Via Campi Flegrei, 34, 80078 Pozzuoli (NA), Italy; irene.bonadies@cnr.it; 2 Elleva Pharma S.R.L. Via Pietro Castellino, 111, 80131 Naples, Italy; francesca.dicristo@ellevapharma.com (F.D.C.); anna.valentino@ellevapharma.com (A.V.); 3 Research Institute on Terrestrial Ecosystems (IRET)—CNR, Via Pietro Castellino 111, 80131 Naples, Italy; gianfranco.peluso@cnr.it (G.P.); anna.disalle@cnr.it (A.D.S.)

**Keywords:** bioresorbable membrane, phytochemicals, resveratrol, polylactic acid, electrospinning, antibiofilm

## Abstract

To date, the implant-associated infections represent a worldwide challenge for the recently reported bacterial drug resistance that can lead to the inefficacy or low efficacy of conventional antibiotic therapies. Plant polyphenolic compounds, including resveratrol (RSV), are increasingly gaining consensus as valid and effective alternatives to antibiotics limiting antibiotic resistance. In this study, electrospun polylactic acid (PLA) membranes loaded with different concentrations of RSV are synthesized and characterized in their chemical, morphological, and release features. The obtained data show that the RSV release rate from the PLA-membranes is remarkably higher in acidic conditions than at neutral pH. In addition, a change in pH from neutral to slightly acidic triggers a significant increase in the RSV release. This behavior indicates that the PLA-RSV membranes can act as drug reservoir when the environmental pH is neutral, starting to release the bioactive molecules when the pH decreases, as in presence of oral bacterial infection. Indeed, our results demonstrate that PLA-RSV2 displays a significant antibacterial and antibiofilm activity against two bacterial strains, *Pseudomonas aeruginosa* PAO1, and *Streptococcus mutans*, responsible for both acute and chronic infections in humans, thus representing a promising solution for the prevention of the implant-associated infections.

## 1. Introduction

Over the past decades, osseointegrated implants have emerged as a major clinical therapeutic approach to replace missing teeth or restore the structure or function of the musculoskeletal system [1]. With the increasing number of implants applied in clinical environments, evidence revealed that the device-associated infections (defined as peri-implantitis) damage the epithelial and mucosal barriers, either impeding host defense mechanisms or serving as microorganism reservoirs, leading to the failure of the implant [2]. Peri-implantitis represents a pathological condition involving both the soft and the hard tissue around implants, characterized by local tissue inflammation that may result in severe bone loss around the implant [3,4]. The inflammatory process involves proliferation of pathogenic bacteria and may occur either shortly after implantation or after several years [3]. The progressive loss of contact between the connective tissue and the implant surface enables the bacterial biofilm to move down into the peri-implant pocket, thereby decreasing implant osseointegration. This process may be exacerbated by the oral pH drop that is a consequence of sugar consumption or sustained microorganism metabolism, as in the biofilm presence [5,6]. Indeed, when the environmental pH reaches values of <5.5, a mineral imbalance between the tooth and the salivary/plaque fluid is generated, resulting in a net tooth loss of hydroxyapatite that blocks the mineralization process [7].

The current non-surgical therapy of peri-implantitis consists of the use of strict aseptic procedures and the administration of systemic antibiotics. However, currently, there is no treatment that acts against the bacteria simultaneously promoting regeneration of the damaged tissue. Additionally, local or systemic administered antibiotics are largely ineffective for peri-implant infections due to bacterial drug resistance, poor drug penetration, and suboptimal bioavailability at the site of infection [2]. Recent studies have demonstrated the effectiveness of plant secondary metabolites (phytochemicals) where bacterial resistance mechanisms, including multidrug resistance, make traditional therapy unsuccessful, even in the control of biofilms [8,9]. In this respect, phytochemicals exert their antibacterial activity through different mechanisms of action including bacterial membrane damage, inhibition of enzymes and toxins, and bacterial biofilm formation. Therefore, plant-derived biomolecules could be used alone or as synergists/potentiators of less effective antibacterial products [8].

Resveratrol (RSV), a stilbenoid polyphenolic compound present in red wine and numerous plants, has demonstrated several health-beneficial effects such as antioxidant, anticancer, anti-inflammatory, and bone regeneration [10]. Numerous studies reported the antimicrobial activity of RSV against a wide range of bacterial [11,12,13,14], viral [15], and fungal species [14] due to the reduction of microorganism motility [16], inhibition of biofilm formation [11], and interference with quorum sensing [17]. Nevertheless, the exact mechanism of the antibacterial and antibiofilm activity of RSV remains uncertain. However, the clinical applications of these results remain controversial, due to RSV poor pharmacokinetics, low water solubility, and in vivo rapid metabolism [18,19]. The local administration into the peri-implant region could address this issue as it helps avoiding systemic degradation of RSV, increasing its therapeutic concentration.

Several studies have been conducted in the area of biomaterials to develop local drug delivery systems that are able to improve the tissue/bone regeneration and treat peri-implant bone infection which limits the osseointegration of the implant [20,21]. In this context, electrospinning represents a simple and cost-effective process to obtain, from a wide range of polymers, drug release devices with high porosity, high surface area, and nanoscale-sized fibers [22]. Electrospun loaded membranes for the treatment of peri-implantitis have been previously reported [23,24]. Li et al. and Zhang et al. fabricated poly(lactic-co-glycolic) acid (PLGA) nanofibers able to release gentamicin and vancomycin, respectively, that can prevent implanted-related infections [21,25]. In particular, PLGA-coated titanium implant with gentamicin, achieving a significant reduction in adhesion of *Staphylococcus aureus* and no cytotoxicity on osteoblasts [21]. In addition, Zhang et al. determine nanofibers with antibacterial properties both in vitro and in vivo against *S. aureus* [25]. Shahi et al. produced tetracycline-containing fibers able to inhibit the growth and the biofilm formation of peri-implantitis-associated pathogens [26]. In particular, membranes manufactured from a polymer blend solution of poly(D,L-lactic acid) (PLA), poly(ε-caprolactone) (PCL), and gelatin (GEL) with different concentrations of tetracycline were obtained. The biofilm reduction was proportional to the tetracycline content. In another research, Baranowska-Korczyc et al. synthesized electrospun PCL membranes loaded with ampicillin that exhibited a good antibacterial activity against an oral strain of *Streptococcus sanguinis* and low cytotoxic effect on gingival fibroblasts [27]. However, to date, no instances of electrospun devices incorporating phytochemicals have been reported for the treatment of peri-implant infections.

In this paper, electrospun PLA nanofiber membranes loaded with different amounts of RSV were produced, characterized, and their antibacterial and antibiofilm potential evaluated on *Pseudomonas aeruginosa* PAO1, *Streptococcus mutans*, and on a mixed culture of both bacteria to simulate the naturally occurring multispecies biofilm system (dual system).

PAO1, one of the most important Gram-negative bacteria, is responsible for both acute and chronic infections in humans. *P. aeruginosa* biofilms were reported to cause several medical device-related infections such as endocardial valve infection through endocardial tubes, ventilator-associated pneumonia, and catheter-associated urinary tract infections [28,29]. Additionally, PAO1 was recovered in patients affected by peri-implant disease in several oral sites [30].

*Streptococcus mutans*, a gram-positive bacterium present in the supragingival region of both healthy people and subjects with periodontal disease, is considered as one of the major contributors in the formation and development of the extracellular polysaccharide matrix in dental biofilms [31]. Indeed, after sucrose consumption, when the oral environment shows a low pH (<5.5) and a strong presence of glycan, bacterial species such as *S. mutans* start to produce water-insoluble glucan, one of the first molecules that significantly contribute to biofilm formation [32]. Then, the glucans synthesized by *S. mutans* provide the substrate for the adhesion of the latecomer bacterial strains [31,33].

The results reported herein demonstrate the ability of the PLA-RSV membranes to release RSV in a tunable and sustained manner, with a release kinetics strongly affected by the pH of the medium. Indeed, the change in pH (from neutral to slightly acidic) triggers a significant increase in the RSV release, demonstrating that the proposed membranes act as pH-responsive RSV reservoirs able to quickly release RSV only in the case of bacterial infection when the pH decreases. Our results, moreover, demonstrate the ability of PLA-RSV membranes to induce a significant antibacterial and antibiofilm activity against PAO1, *S. mutans*, and a mixed culture of both bacteria at pH < 5.5.

Taken together, the reported data suggest that PLA-RSV membranes can represent a promising solution for the prevention of the implant-associated infections, both as barrier membranes during a socket preservation period and as implant coating for prolonged time use.

## 2. Materials and Methods

### 2.1. Materials

Polylactic acid (PLA, Ingeo 4032D) with 0.7 mol% L-isomer, Mw = 2.1 × 10^5^ g mol^−1^, and the polydispersity (PDI) = 1.7 were supplied by NatureWorks LLC (Minnetonka, Minnesota, USA). *N,N*-Dimethylformamide (DMF), acetone with a purity of ≥99.8%, and resveratrol (RSV) were purchased from Sigma-Aldrich (Milan, Italy) and used without further purification.

### 2.2. Preparation of Electrospinning Solutions and Membrane Manufacturing

Electrospun membranes containing resveratrol were realized starting from PLA solutions containing different amounts of resveratrol. Neat PLA solutions (coded as PLA) were prepared by dissolving 12.5% wt. PLA in acetone/DMF (80/20 v/v) at 60 °C; after that, 0.8% and 3.2% wt of RSV with respect to PLA (w/w) were directly added to the polymer solution (coded as PLA-RSV1 and PLA-RSV2, respectively). The solutions were stirred before use for at least 6 h at 60 °C. All the solutions were electrospun with a NANON01 equipment (MECC Co., Ltd., Fukuoka, Japan) by using a single nozzle and a plate collector at room temperature and 10% relative humidity. After optimization of the process parameters, the flow rate was fixed at 0.5 mL h^−1^. The applied voltage and the distance between the nozzle and the collector, which was covered with aluminum foil, were adjusted to 20 kV and 30 cm, respectively, to obtain defect-free fibers for further characterizations.

### 2.3. Membranes Characterization

The morphology of the membranes was evaluated using a FEI Phenom Desktop Scanning Electron Microscope (Eindhoven, The Netherlands). Before analysis, the samples were sputtered/coated with an Au-Pd alloy using a Baltec Med 020 Sputter Coater System (Leica, Milan, Italy) and then mounted on aluminum stubs. The average fiber-diameter distribution was analyzed using the ImageJ software 1.51 April 2018 (NIH, Bethesda, MD, USA).

The chemical composition of membranes was investigated by means of Fourier Transformed Infrared Spectroscopy coupled with attenuated total reflectance technique (ATR-FTIR). The spectra were acquired in the spectral region between 4000 and 400 cm^−1^. The analysis was performed using the Origin software (OriginPro 8.1 SR0, 2009 October, OriginLab Corporation, Northampton MA, USA). Resveratrol spectrum was considered as positive control.

### 2.4. In Vitro Drug Release from RSV-Loaded Membranes

The RSV release was investigated as reported by Riccitiello et al. with some modifications [34]. Briefly, circular pieces of membranes (30 mm diameter) were weighed and placed into individual vials covered with aluminum foil to prevent drug degradation caused by light. The release kinetics was performed at 37 °C in artificial saliva medium containing 4 g of sucrose (SAGF-suc), as reported by Cavazana et al. [5]. The pH was adjusted to 4.8 and 6.8 with HCl and NaOH, respectively. At predetermined time intervals (every hour for 24 h, then every 3 days over 90 days), supernatants were withdrawn, and the same amount of fresh solution was added back to the release medium to maintain the sink condition. The RSV concentration was measured using HPLC-UV with a linear elution gradient consisting of mobile phase A (0.1% acetic acid), B (Acetonitrile), and C (Methanol). The detection wavelength was set at 290 nm and RSV quantitation was based on a standard curve in SAGF-suc. The resveratrol stock standard of 1 mg/mL was prepared in methanol. Before injection, the standards and samples were filtered through a 0.22 μm pore-size filter (Millipore, Milan, Italy). System control and data acquisition were performed using the ChemStation software 4.03 Jan 27, 2020 (Agilent Technologies). The results were presented in terms of cumulative release as a function of time.

### 2.5. Bacterial Strains and Culture Conditions

*Pseudomonas aeruginosa* PAO1 (ATCC^®^ BAA-47™) and *Streptococcus mutans* (ATCC^®^ 25175) were obtained from the American Type Culture Collection (ATCC, LGC Standards S.R.L., Sesto San Giovanni, Milan, Italy), and cultivated following the ATCC guidelines. Briefly, PAO 1 and *S. mutans* were cultured for 18 h on trypticase soy broth agar and trypticase soy yeast extract agar (Thermo Fisher Scientific, Waltham, MA, USA), respectively. Subsequently, one colony was resuspended in 5 mL of liquid broth medium and incubated overnight at 37 °C and 200 rpm.

### 2.6. Antibacterial Activity

The capability of the RSV-loaded membranes to inhibit bacterial growth was assessed by monitoring the bacterial growth rate. Each electrospun membrane of similar dimension, previously sterilized by UV radiation for 15 min at each side, was placed in a 12-well plate, covered with 500 µL of liquid broth supplemented with 20% of sucrose [5], and inoculated with a bacterial suspension containing a microbial concentration of approximately 1 × 10^7^ CFU/mL. The plate was incubated at 37 °C and 200 rpm in a microplate reader (Cytation 3; AHSI, Milan, Italy). At scheduled times (6 h, 24 h, or 48 h), the optical density (OD) at 600 nm was recorded. Moreover, culture pH was monitored using a pH electrode (Mettler-Toledo, Milan, Italy).

### 2.7. Biofilm Analysis

Biofilm was developed as described by Di Salle et al. with some modifications [35]. Briefly, each electrospun membrane of similar dimension was sterilized by UV radiation for 15 min at each side. Then, the membranes were placed in a 48-well polystyrene plate, covered with 750 µL of liquid medium broth supplemented with 20% of sucrose and containing *S. mutans*, PAO1 or a mixed culture of both bacteria (PAO1-*S. mutans*), with a concentration of 1 × 10^7^ CFU/mL. The cultures were incubated statically at 37 °C in a humid atmosphere for 16 h, until a mature biofilm was obtained. Liquid medium broth without bacteria was used as negative control, while 750 µL of PAO1 (1 × 10^7^ CFU/mL), *S. mutans* (1 × 10^7^ CFU/mL), PAO1-*S. mutans* (1 × 10^7^ CFU/mL each), and 200 µM resveratrol were used as positive controls.

Crystal violet (CV) assay was used to determine biofilm formation, as previously described [35]. Briefly, each well was washed twice with sterile PBS for removal of non-attached bacteria, air-dried for 15 min, stained with 0.1% w/v crystal violet for 30 min, and then re-washed with 5 × PBS to remove any extra stain. Then, the stained biofilms were solubilized in 96% ethanol and absorbance measured at 570 nm using a microplate reader (Cytation 3, AHSI, Milan, Italy).

The number of biofilm viable bacterial cells was determined with the BacTiter-Glo™ Microbial Cell Viability Assay (Promega, Milan, Italy) and with the Live/Dead Cell Double Staining Kit (Sigma Aldrich, Milan, Italy). The tests were performed following the manufacturer’s protocol. The BacTiterGlo™ assay is based on the luciferase reaction driven by ATP released from lysed bacterial cells. Therefore, to quantify the ATP present in the culture, 250 µL of PBS and 250 µL of BacTiter-Glo™ Reagent were added to each well, mixed, and incubated at room temperature for 5 min. The luminescence as relative light units (RLU) was measured in a microplate reader (Cytation 3, ASHI, Bernareggio, Italy) and correlated with the number of metabolically active bacteria, using an ATP calibration curve.

Staining with the LIVE/DEAD^®^ Biofilm viability kit (Molecular Probes, Life Technologies Ltd., Milan, Italy) was performed according to the manufacturer’s instructions. Briefly, a working solution of fluorescent stains was prepared by adding 3 μL of SYTO^®^ 9 stain and 3 μL of propidium iodide (PI) stain to 1 mL of filter-sterilized water. Two hundred microliters of staining solution were deposited on the disc surface and, after 15 min incubation at room temperature in the dark, samples were washed with sterile saline in order to remove the excess dyes and rinsed with water from the base of the support material. Fluorescence was detected using Cytation 3 with 490 nm excitation for simultaneous monitoring of viable and dead cells. Measurements were carried out in triplicate for each membrane.

### 2.8. Statistical Analysis

All quantitative data are presented as the mean ± SD. Each experiment was performed at least three times. Student’s *t* test was used for the resveratrol release. Statistical analyses for the antibacterial and antibiofilm assays were performed by 1-way analysis of variance (ANOVA) with Bonferroni’s post hoc test. All the data were analyzed with the GraphPad Prism version 8.01 statistical software package (GraphPad, CA, USA).

## 3. Results

### 3.1. Membrane Characterization

Nowadays, biodegradable polymers such as aliphatic polyesters have replaced traditional non-degradable materials for biomedical application due to their ability to degrade and be absorbed by the body without elicit adverse effects [36,37]. Among them, electrospun PLA-based nanofibers represent one of the most promising drug release systems because of their superior chemical and mechanical properties, their versatility in fabrication, biodegradability, and compatibility with biomolecules and cells [38]. Riccitiello et al. fabricated uniform defect-free fibers of PCL and PLA that were able to release RSV in a tunable and sustained manner. Both membranes showed similar in vitro osteoinductive capacity on dental pulp stem cells, while the lower resveratrol-releasing membrane (PLA-RSV) was able to inhibit osteoclast differentiation [34]. The bactericidal properties of PLA membranes with high loadings of titanium dioxide nanoparticles (TiO_2_) were reported by Toniatto and co-workers [39]. In addition, the nanostructured PLA/TiO_2_ nanofibers demonstrated no mammalian cell toxicity, suggesting a wide range of biomedical applications.

To evaluate the effect of release media on membrane fiber morphology, stability, and release kinetic, PLA membranes with two RSV concentrations (PLA-RSV1 and PLA-RSV2) were characterized before and after the in vitro RSV release test.

**Figure 1:**
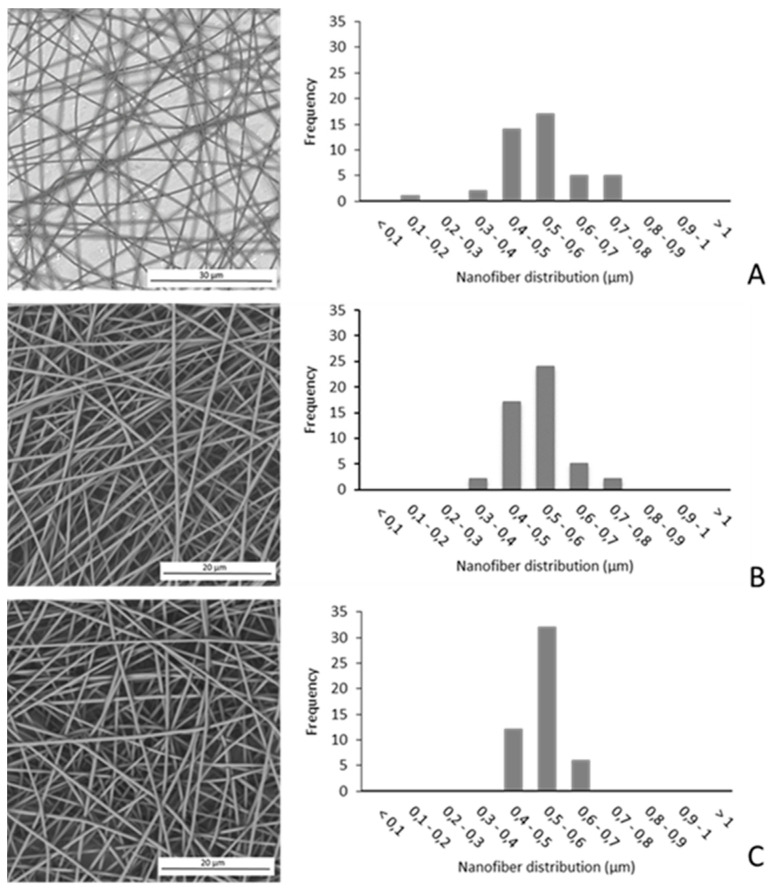
SEM micrographs (left) and size-distribution of fibers (right), expressed as % number (frequency), prepared from polylactic acid (PLA) solutions containing various amounts of resveratrol (RSV): (**A**) PLA; (**B**) PLA-RSV1; (**C**) PLA-RSV2.

SEM micrographs and diameters distribution of the PLA-RSV fibers containing different amounts of RSV are shown in Figure 1. All membranes exhibited a three-dimensional interconnected pore structure. For all analyzed compositions, fibers have a smooth and regular surface, with a uniform bead-free diameter, and no appearance of drug aggregates. A monomodal distribution was observed for all samples; the addition and the amount of RSV did not affect the average diameter of fibers that was 0.540 ± 0.103, 0.531 ± 0.075, and 0.545 ± 0.054 µm for PLA, PLA-RSV1, and PLA-RSV2, respectively.

**Figure 2:**
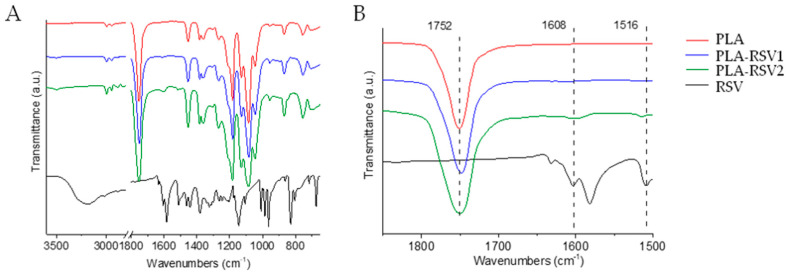
(**A**) FTIR-ATR spectra of RSV, neat PLA fibers, PLA fibers containing RSV1 and RSV2; (**B**) Zoom in the range of 2000–1400 cm^−1^.

FTIR analysis of PLA and PLA-RSV membranes revealed the characteristic peaks of both RSV and PLA (Figure 2). Despite several vibration bands of PLA and RSV overlapping, it was possible to highlight the vibration of phenol OH at 3505 cm^−1^, the C=C aromatic double bond stretching related to the aromatic rings of RSV at 1608 cm^−1^, the C–C olefinic stretching at 1599 cm^−1^, and the in-plane C−H bending of phenyl rings at 1516 cm^−1^. The intensity of RSV peaks increased with the amount of RSV in the fibers. Furthermore, peaks at 1749 cm^−1^ (C=O stretching) and 1453 cm^−1^ (–CH_3_ bending) related to PLA were evident [40,41]. Interestingly, these peaks shifted to lower wavenumbers in comparison with the same bands in the pure RSV (3201, 1605, 1583, and 1510 cm^−1^) and PLA (1752 cm^−1^) spectra, indicating the presence of hydrogen-bonding interactions between the phenol OH of RSV and the carbonyl oxygen of the PLA matrix [42].

### 3.2. pH-Dependent RSV Release

The local delivery of a bioactive molecule from the electrospun membrane is significantly influenced by the polymer–drug interaction and the physicochemical properties of the membrane, such as drug encapsulation efficiency, drug distribution inside the micro/nanofibers, and drug release kinetics [43,44].

**Figure 3:**
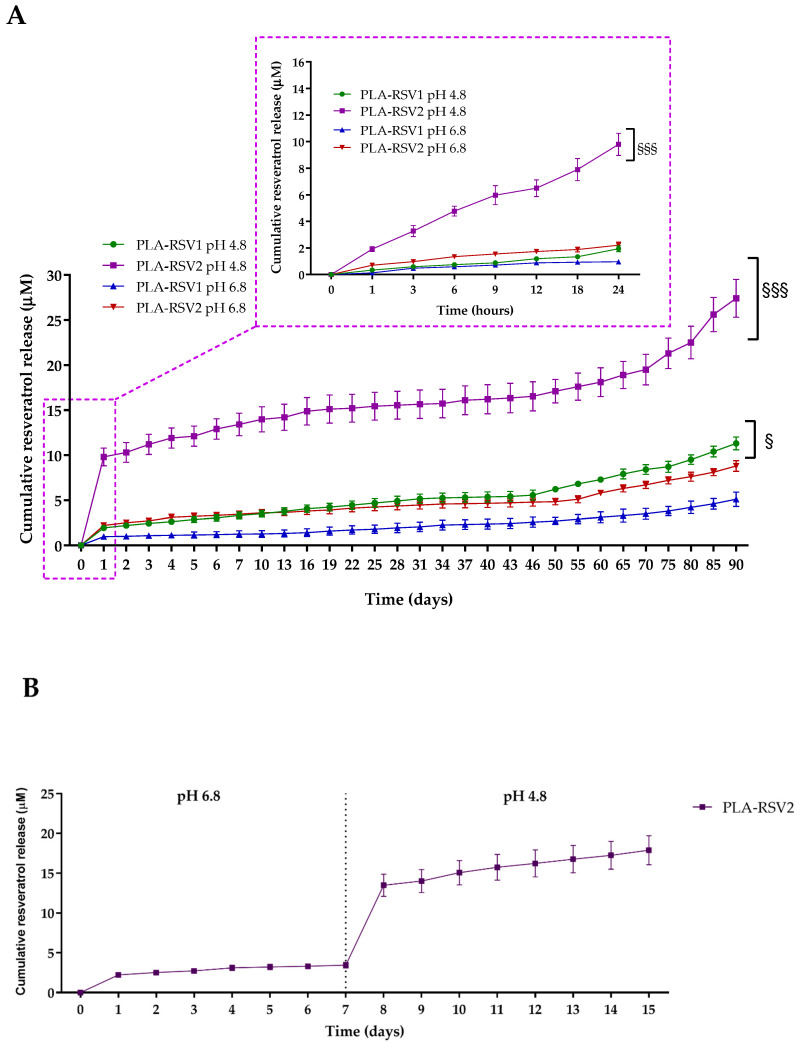
(**A**) In vitro drug release profiles at 37 °C of PLA-RSV membranes incubated for 46 days in artificial saliva supplemented with sucrose (SAGF-suc) at pH 4.8 and pH 6.8. (**B**) In vitro drug release profiles of PLA-RSV2 membrane in SAGF-suc at pH 6.8 from 0 to 7 days and at pH 4.8 from 8 to 15 days. For each sample, six different experiments were conducted, and the results expressed as the mean of the values obtained (mean ± SD). Statistically significant variations: §§§ *p* < 0.001 and § *p* < 0.05 versus pH 6.8.

In particular, the mechanism of drug release from polyester nanofibers is characterized by a two-phase release behavior: first, the embedded compound desorbs through fiber nanopores or from the outer surface of the fibers in contact with the medium, then the drug captured between polymer chains is released [45,46]. To mimic the peri-implant microenvironment, characterized by subtle variations of pH level or degree of inflammation, in vitro cumulative release of RSV from PLA membranes was performed at different pH conditions, namely, pH 4.8 and pH 6.8, in artificial saliva supplemented with sucrose (SAGF-suc). As shown in Figure 3A,B, a burst release was noticed on the first day, suggesting that RSV located in the outer surface of the fibers quickly diffuses in the medium [47]. It is worth noting that in acidic conditions the RSV release rate from the PLA-RSV membranes was remarkably higher than that at neutral pH. In particular, after one day of incubation at pH 4.8, the RSV release for PLA-RSV1 and PLA-RSV2 was two- and five-fold higher, respectively, than that at pH 6.8. Subsequently, a slow but sustained release continued at pH 4.8 for 46 days, reaching a concentration of 5.57 ± 0.56 and 16.52 ± 1.62 μM at that time for PLA-RSV1 and PLA-RSV2, respectively. Instead, at neutral pH, the RSV amount released from PLA-RSV1 and PLA-RSV2 was approximately 50% and 75% lower than that released at pH 4.8 in 46 days highlighting that the effect of pH was more significant in the case of higher RSV loading. Furthermore, while 50% of RSV was released from PLA-RSV2 at the end of 46 days at pH 4.8, only 14.9% was released in the same time at pH 6.8. To better understand the impact of the pH on the RSV release, the PLA-RSV2 sample was first immersed in SAGF-suc pH 6.8 for 7 days and then transferred to pH 4.8. As shown in Figure 3B, the change in pH led to a sudden increase (~3.9-fold, *p* < 0.001) in the RSV concentration, due to the faster release of RSV entrapped in the fiber surface. It should be also noted that the slope of the release curve at pH 4.8 was about three times higher than that at pH 6.8, as the acidic pH also induced faster release of the drug captured in the bulk polymer fiber. The results demonstrate that the membranes can be stored for several days at physiological pH before RSV is quickly released when the pH decreases, as in the case of bacterial infection.

**Figure 4:**
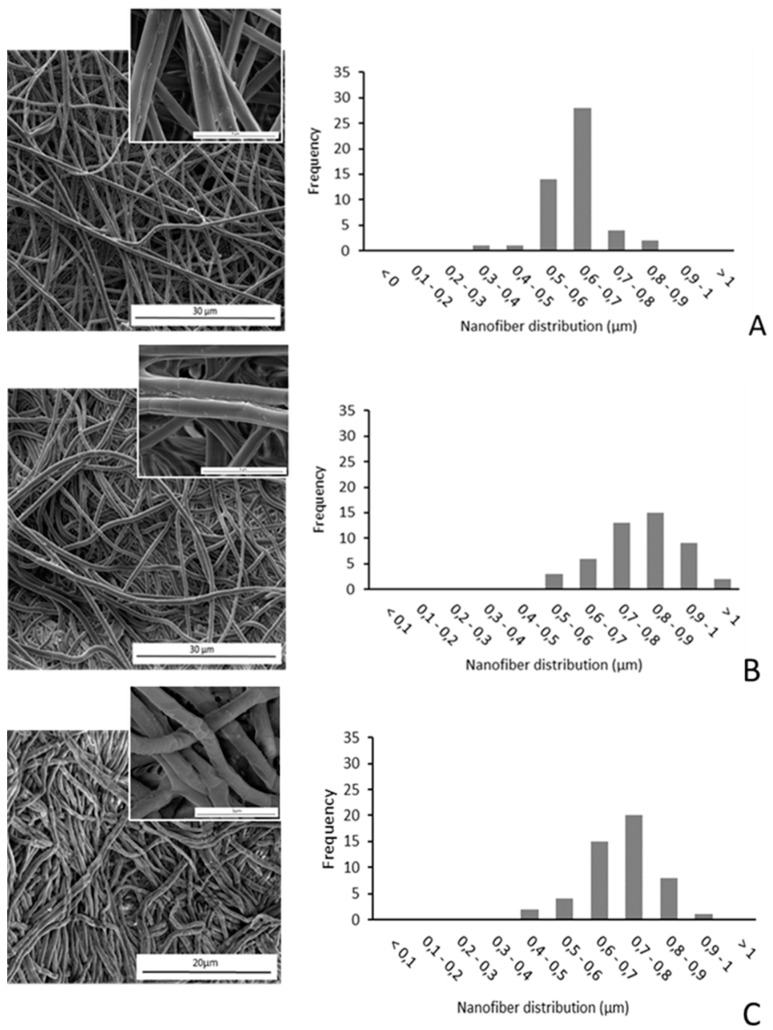
SEM micrographs (left) and size-distribution of fibers (right), expressed as % number (frequency), prepared from PLA solutions containing various amounts of RSV after a 46-day release test: (**A**) PLA-RSV1, pH 4.8, (**B**) PLA-RSV2, pH 4.8, (**C**) PLA-RSV2, pH 6.8.

The morphological analysis carried out on the samples after the release test reveals no massive deterioration of fibers even after 46 days incubation for both pH values (Figure 4). A close inspection of the polymer surface after 46 days incubation (inset in Figure 4) revealed massive fiber swelling, which led to an increase in fiber diameter proportional to the amount of RSV, and remarkably affected by the pH of the release medium.

**Figure 5:**
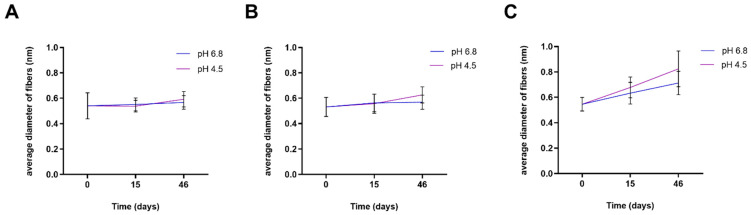
Average diameters of fibers prepared from PLA solutions containing various amounts of RSV after incubation in SAGF-suc (pH 4.5 and pH 6.8) for 15 and 46 days: (**A**) PLA, (**B**) PLA-RSV1, and (**C**) PLA-RSV2.

As shown in Figure 5, in the case of PLA-RSV2, the immersion for 46 days at pH 6.8 and 4.8 resulted in a diameter increase by about 31% and 51%, respectively. This increase in nanofiber diameters is due to the swelling process occurring during incubation in SAGF-suc for the release test. For PLA and PLA-RSV1 the diameter variation resulted negligible at both pH examined. As reported in literature, soaking of the nanofibers allows the water molecules to penetrate into the fiber interior, resulting in the swelling process. Remarkably, acidic conditions significantly enhance the ability of water to penetrate the polymer, giving rise to a higher swelling ratio [48,49].

**Figure 6:**
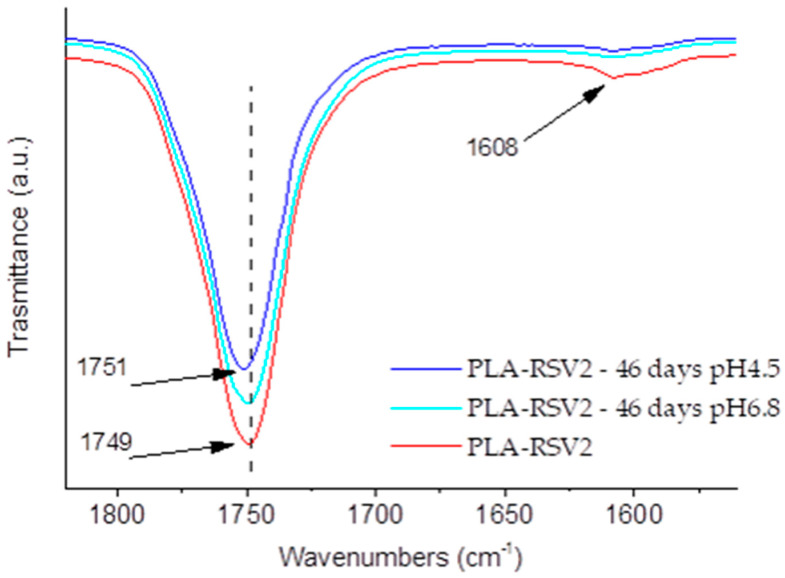
ATR spectra of RSV, neat PLA fibers, and PLA fibers containing RSV2 after the release test (pH 4.5 and pH 6.8—46 days) in the range of 1820–1560 cm^−1^.

FTIR analysis of PLA and PLA-RSV membranes after incubation in the release medium at different pH values provides further insights about the release mechanism of the membranes (Figure 6). First, the decrease of the RSV peak at 1608 cm^−1^ confirms that RSV is released from the fibers. Further, a slight shift of the C=O absorption band to higher wavenumbers, from 1749 to 1751 cm^−1^ for PLA-RSV2 at pH 4.5, was noticed after the 46-day immersion tests in the release medium, confirming the disruption of the hydrogen bonding between RSV hydroxyl and PLA carbonyl groups.

Taking into account the results gathered from SEM observations and FTIR spectroscopy, and considering that in the experimentally used pH conditions the influence of pH on the PLA degradation rate is negligible [50,51,52], the high RSV release is ascribed to the activity of the water molecules that provide the driving force for the RSV diffusion as the membrane swells in acidic conditions [53]. Additionally, a slightly higher RSV solubility in acidic media can further contribute to the enhancement of the RSV release at pH 4.5 [54]. It is also worth noting that for longer incubation times (Figure 3A) the shape of the release curve exhibited a significant upward deviation at times exceeding 50 days. This outcome suggests the occurrence of polymer degradation, thereby resulting in a further acceleration of the RSV release. To sum up, the overall kinetics of the RSV release was characterized by a three-phase profile. At the very beginning, the compound embedded in the outer surface quickly desorbs from the fibers. Subsequently, the drug captured in the core diffuses out of the fibers [55]. In this regard, the acidic pH enhances the water-induced swelling, which is the main reason responsible for the RSV release. Afterwards, the effect of polymer degradation on RSV release becomes more significant, giving rise to a further acceleration of RSV diffusion. It should be also noted that the effect of pH was more significant in the case of higher RSV loading.

### 3.3. Antibacterial and Antibiofilm Activity

Phytochemicals may represent a valid alternative or adjunctive to antibiotics for mitigating implant-related infections, thanks to their reduced risk of developing resistant bacterial strains [56,57]. In addition, plant-derived compounds may exert in vitro synergistic effects when combined with conventional antibiotics [58]. Phenolic compounds, such as resveratrol, play an important role in enhancing antibiotic activity against resistant pathogens reducing, for example, the efflux pumps activity or acting as an employing EP inhibitors (EPIs) strand [14,59]. Phenolic terpenes such as carvacrol, thymol, and geraniol have been found to exhibit marked antibiofilm activity against both fungal and bacterial biofilms encountered in food processing environments and biomedicine [60,61]. Furthermore, several studies also reported the efficacy of resveratrol in the inhibition of formation and elimination of both Gram-positive and Gram-negative bacterial biofilm [13,62,63].

The capability of RSV-loaded PLA fibers to inhibit bacterial growth was determined by monitoring the growth rate of *S. mutans*, PAO1, and a mixed culture of both bacteria (PAO1-*S. mutans*) at 600 nm. The growth medium was supplemented with 20% of sucrose to mimic clinical conditions related to dental diseases such as periodontal diseases. Several studies, indeed, indicated how the presence of sugars in the diet increases the bacterial acid lactic production that lead to an environmental pH decrease [5,7].

The pH of the culture medium was measured at the beginning of the test, and after specific time-points throughout the experiment. It was found that pH reached acidic values already after the first 6 h of growth (Table 1).

**Figure 7:**
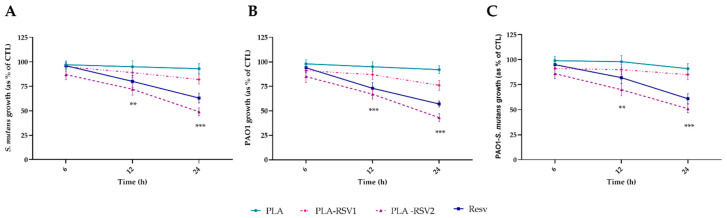
Antibacterial activity evaluated at 600 nm against *Streptococcus mutans* (**A**), *Pseudomonas aeruginosa* PAO1 (**B**), and PAO1-*S. mutans* (**C**) of PLA, PLA-RSV1, and PLA-RSV2. Bacterial growth in absence of membranes was used as bacterial positive control (CTL) while 200 µM resveratrol was used as positive standard control (RSV). Data were reported as a percentage in comparison with a bacterial positive control. For each sample, six different experiments were conducted and the results expressed as the mean of the values obtained (mean ± SD). Statistically significant variations: ** *p* < 0.01, and *** *p* < 0.001 versus PLA and PLA-RSV1.

As shown in Figure 7, the PLA-RSV2 membrane was able to significantly inhibit bacterial growth already after 12 h of incubation (*p* < 0.01). In particular, the greatest effect was observed on *Pseudomonas aeruginosa* at 24 h, showing a 57% of growth inhibition with respect to the control (*p* < 0.001). No effect was observed in the presence of PLA-RSV1.

**Figure 8:**
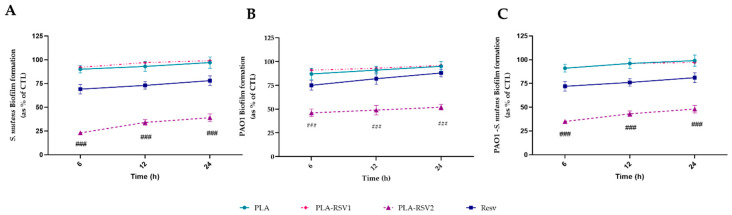
Antibiofilm activity of RSV-loading membranes. Biofilm formation was evaluated by crystal violet (CV) assay, after 6, 12, and 24 h of incubation at 37 °C in presence of (**A**) *Streptococcus mutans*, (**B**) PAO1, and (**C**) PAO1-*S. mutans* as described in the material and methods section. Biofilm formation was reported as a percentage in comparison with to the maximum amount of biofilm produced by *Streptococcus mutans*, PAO1, and PAO1-*S. mutans* grown (bacterial positive controls). A total of 200 µM resveratrol was used as positive standard control (RSV). For each sample, six different experiments were conducted, and the results expressed as the mean of the values obtained (mean ± SD). Statistically significant variations: ### *p* < 0.001 versus RSV, PLA, and PLA-RSV1.

Biofilm inhibition was analyzed at different times in order to evaluate the resveratrol effect on biofilm development. As shown in Figure 8, a significant reduction (*p* < 0.001) in biofilm formation analyzed with CV assay, regardless of the bacterial strain used, was observed only in PLA-RSV2 membrane. Already after 6 h of incubation in presence of the membrane, a reduction of about 77 ± 8%, 60 ± 6%, and 65 ± 6% was detected in *S. mutans*, PAO1, and PAO1-*S. mutans* biofilm, respectively.

Metabolic analysis of biofilm, performed quantifying the amount of cellular adenosine triphosphate (ATP) that is directly related to bacterial metabolic activity [64], confirmed the CV results.

**Figure 9:**
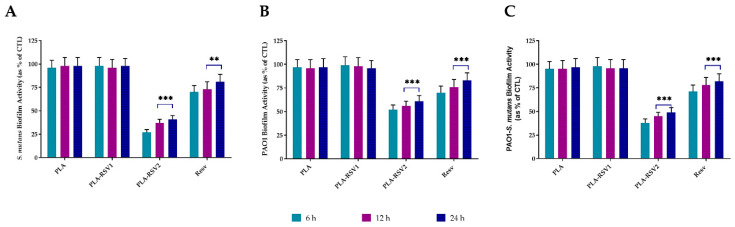
Biofilm metabolic activity of RSV-loading membranes. Biofilm metabolic reduction was quantified using BacTiterGlo™ assay and correlating the recorded luminescence with APT nmoles, after 6, 12, and 24 h of incubation at 37 °C in presence of (**A**) *Streptococcus mutans*, (**B**) PAO1, and (**C**) PAO1-*S. mutans* as described in the material and methods section. Biofilm activity was reported as a percentage in comparison with positive bacterial controls. A total of 200 µM resveratrol was used as positive standard control (RSV). For each sample, six different experiments were conducted, and the results expressed as the mean of the values obtained (mean ± SD). Statistically significant variations: ** *p* < 0.01 and *** *p* < 0.001 versus PLA and PLA-RSV1.

Figure 9 shown that PLA-RSV2 membrane was able to reduce biofilm activity already at 6 h and maintaining its activity until 24 h. The observed reduction after 6 h of incubation ranged from 27 ± 3% for *S. mutans* biofilm to 52 ± 5% for PAO1 biofilm.

The susceptibility of *S. mutans* and *P. aeruginosa* to RSV was further evaluated via the Live/Dead BacLight Bacterial Viability Kit. The fluorescent dye Syto9 (green) penetrates bacterial membrane of both live and dead bacteria, while propidium iodide (red) selectively enters damaged bacteria membrane allowing differentiation between live and dead cells [65].

**Figure 10:**
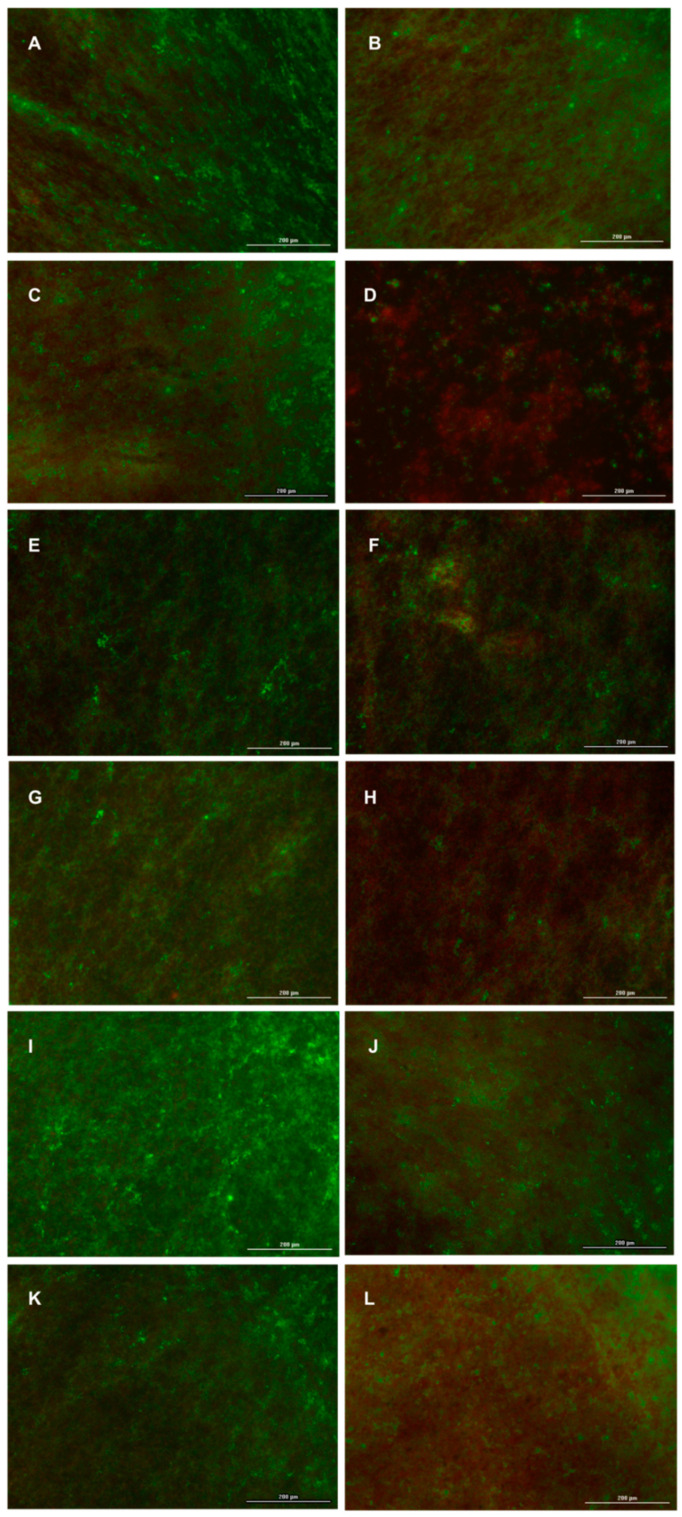
Fluorescent microscopy images of live/dead staining of (**A**–**D**) *S. mutans*, (**E**–**H**) PAO1, and (**I**–**L**) *S. mutans*/PAO1 on (**A**,**E**,**I**) PLA, (**C**,**G**,**K**) PLA-RSV1, and (**D**,**H**,**L**) PLA-RSV2. RSV was used as positive control (**B**,**F**,**J**). Live bacteria were stained green, and dead bacteria were stained red. Live and dead bacteria in proximity resulted in yellow/orange color.

As shown in Figure 10A,E,I, PLA membrane exhibited a well-organized, undisturbed biofilm with a lower live/dead cell ratio indicative of a general bacterial population in stationary phase growth. According to the BacTiterGlo™ results, there were no significant changes in viability of biofilm formed by *Streptococcus mutans* (Figure 10C), PAO1 (Figure 10G), and PAO1-*S. mutans* (Figure 10K) on PLA-RSV1 membranes in regards to PLA. In contrast, the biofilm formed by *S. mutans* or PAO1*-S. mutans* on PLA-RSV2 membrane (Figure 10D,H) presents a lessened biofilm mass/architecture with a significant proportion of dead cells confirming the antibacterial activity of the manufactured membrane. In the case of PAO1 (Figure 10L), an overlap between red and green was observed (labeled as yellow), which indicates the initial stages of apoptotic processes. Biofilm treated with 200 µM RSV exhibited a higher live/dead cell ratio in regards to PLA membrane for all the microorganisms analyzed (Figure 10B,F,J).

Thus, these qualitative findings further confirmed that the newly fabricated PLA-RSV2 membrane possessed good antibiofilm activity inducing a cell membrane damage.

Taken together, these results indicated that the PLA-RSV2 membrane was able to control the biofilm formation process inhibiting the bacterial ability to reproduce and form a mature biofilm during the 24 h. Indeed, PLA-RSV2 released a resveratrol concentration capable of producing a significant antimicrobial and antibiofilm effect already after 6 h of incubation.

## 4. Conclusions

In this paper, electrospun PLA nanofiber membranes loaded with different amounts of RSV were produced, characterized, and their antibacterial and antibiofilm potential evaluated on *Pseudomonas aeruginosa* PAO1 and *Streptococcus mutans* responsible for both acute and chronic infections in humans. The reported results demonstrated that resveratrol released from PLA-RSV2 membrane was able to induce, already after 6 h, a significant decrease of both bacterial growth and biofilm formation. Moreover, the PLA-RSV membranes capability to release RSV only at pH < 5.5 (as in presence of oral bacterial infection) acting as drug reservoir when the environmental pH is neutral, represents an interesting solution in the prevention of implant-associated infections. For this purpose, the PLA-RSV membranes could be used both as barrier membranes during socket preservation period and as implant coating for prolonged time use, taking advance of their pH-responsive release ability.

## Figures and Tables

**Table 1:** pH of the bacterial cultures.

Time	PAO1	*S. mutans*	PAO1-*S. mutans*
6 h	4.82 ± 0.26	4.86 ± 0.51	4.83 ± 0.55
12 h	4.87 ± 0.51	4.80 ± 0.46	4.85 ± 0.53
24 h	4.88 ± 0.43	4.81 ± 0.35	4.84 ± 0.39
